# CXCL10 serves as a potential serum biomarker complementing SCC-Ag for diagnosing cervical squamous cell carcinoma

**DOI:** 10.1186/s12885-022-10142-0

**Published:** 2022-10-08

**Authors:** Jingya Zhang, Dong Dong, Qian Wei, Li Ren

**Affiliations:** Department of Clinical Laboratory, Tianjin Medical University Cancer Institute and Hospital, National Clinical Research Center for Cancer, Tianjin’s Clinical Research Center for Cancer, Key Laboratory of Cancer Prevention and Therapy, Tianjin, Tianjin 300060 P.R. China

**Keywords:** Biomarker, SCC-Ag, Cervical squamous cell carcinoma, CXCL10

## Abstract

**Background:**

Cervical squamous cell carcinoma (CESC) is the most common histological type of cervical cancer which is the major cause of death in women worldwide. Although squamous cell carcinoma antigen (SCC-Ag) is widely used to detect CESC, it is not sensitive and specific enough to predict the disease.

**Methods:**

We investigated serum CXC motif chemokine 10 (CXCL10) as potential diagnostic biomarker in detecting CESC in this study. Serum levels of CXCL10 and SCC-Ag were measured by ELISA or automated immunoassay in 345 participants, including 189 patients with different stages of CESC, 75 patients with cervical intraepithelial neoplasia, and 81 healthy individuals. Performances of CXCL10 and SCC-Ag as single biomarkers were analyzed by the ROC curves. The changes of serum levels of CXCL10 and SCC-Ag in 10 longitudinal followed-up CESC patients with partial response (PR) during chemoradiotherapy or chemotherapy were evaluated.

**Results:**

The two markers showed similar diagnostic capacity in distinguishing both CESC early stage from healthy controls (AUCCXCL10 = 0.740, AUCSCC-Ag = 0.710) and all CESC from healthy controls (AUCCXCL10 = 0.775, AUCSCC-Ag =0.793). Moreover, CXCL10 showed ability in distinguishing cervical intraepithelial neoplasia from healthy control (AUCCXCL10 = 0.727) and cervical cancer SCC-Ag-negative from healthy control. (AUCCXCL10 = 0.739). The combination of CXCL10 and SCC-Ag displayed significant improvement of AUCs than individual SCC-Ag or CXCL10 in the analysis groups (healthy vs all cervical cancer, healthy vs cervical cancer early stage*)*. The AUCs were improved to 0.877 (AUCSCC-Ag = 0.793, *P* < 0.05) to distinguish healthy controls from all CESC and 0.828(AUCSCC-Ag = 0.710, *P* < 0.05) to distinguish healthy controls from CESC early stage by the combination of the two markers, respectively. Significant differences of serum CXCL10 levels were found between CESC patients at late tumor stage and CESC patients at early tumor stage (*P* < 0.01). Serum CXCL10 levels of the CESC patients who had partial response after treatment significantly decreased during treatment (*P* = 0.013), whose consistent and inconsistent frequency with the response were the same as serum SCC-Ag levels.

**Conclusions:**

The results indicated that CXCL10 is a potential serum biomarker complementing SCC-Ag in prediction of CESC. CXCL10 showed ability in the diagnosis of SCC-Ag negative CESC and the combination of CXCL10 and SCC-Ag inhibited improved performance compared with SCC-Ag alone.

**Supplementary Information:**

The online version contains supplementary material available at 10.1186/s12885-022-10142-0.

## Introduction

Cervical cancer ranks as the fourth most common malignancy in women worldwide and the second prevalent gynecological tumor worldwide [1]. Despite the incidence and mortality of cervical cancer have declined due to the improvement of disease screening and application of vaccines in high-risk populations [2, 3], it is still one of the leading causes of deaths in women all over the world [1]. Due to the lack of apparent early symptoms, early detection remains extremely difficult and poor outcomes often occur in the advanced stage [4, 5]. The major treatments such as radiotherapy and chemotherapy are often ineffective in the patients with metastasis and recurrence of cervical cancer [6]. Thus, the best chance for curative treatments and improved outcomes attributes to early diagnosis of cervical cancer.

There are already established tests like Pap smear, ThinPrep cytologic test, acetic acid (VIA) and Lugol’s iodine (VILI) for cervical cancer screening. Pap smear and ThinPrep cytologic test are frequently used for cervical cancer detection with disadvantages of invasiveness and inconvenience. The results of all these methods vary widely due to the subjective understanding and experience of the operators [7–9]. The utility of HPV test in diagnosing cervical cancer is limited for the unsatisfactory specificity because many HPV infections may not process to cervical cancer [10]. Squamous cell carcinoma antigen (SCC-Ag) has been widely used as serum biomarker to identify cervical squamous cell carcinoma (CESC), which is the most common histological type of cervical cancer [11]. However, it is not sufficiently sensitive or specific for early-stage CESC detection. Therefore, it is crucial to find a novel non-invasive and repeatable serum biomarker in diagnosing cervical cancer.

We found that CXCL10 was a potential new circulating biomarker for CESC by analysis of publicly available expression data in the TCGA database (https://tcga-data.nci.nih.gov/tcga/). CXC motif chemokine 10 (CXCL10), also known as interferon-γ-inducible protein 10, is a member of the CXC chemokine family, which are soluble small molecule secretory proteins [12]. CXCL10 may be associated with tumor development and metastasis. The overexpression of circulating CXCL10 was detected in some of human cancers, such as breast cancer and colorectal cancer [13, 14].

However, no data is available regarding serum CXCL10 levels in patients of cervical cancer to date. In this study, we focused on the clinical research for the potential value of serum CXCL10 as a diagnosing biomarker for CESC. We investigated serum CXCL10 levels to evaluate the individual and combined diagnostic performances of CXCL10 and SCC-Ag for CESC. The diagnostic ability of CXCL10 for SCC-Ag-negative CESC was also evaluated. We also tracked CXCL10 and SCC-Ag dynamics of CESC patients with partial response.

## Materials and methods

### Patients and specimens

The serum samples analyzed in this study were obtained from 189 patients with CESC at different stages, 75 patients with cervical intraepithelial neoplasia (CIN) and 81 healthy controls without any malignant diseases at Tianjin Medical University Cancer Institute and Hospital (Tianjin, China) between February 2021 and December 2021. The patients with acute inflammatory diseases, systemic inflammatory diseases were excluded from this study. We also excluded the patients who had other types of cancer, cervical disease or received anticancer therapy before. The study was approved by the Research Ethics Committee of Tianjin Medical University Cancer Institute and Hospital and confirmed to the 1964 Helsinki Declaration ethical standards. Written informed consent was obtained from all the patients and healthy individuals in this study.

Serum samples were collected at baseline or prior to each cycle of treatment (chemoradiotherapy and chemotherapy). The patients who had a minimum of 3 evaluable serial samples were considered as evaluable patients. Thirty subjects with CESC were included for assessing the curative effect, and 13 evaluable patients had several months of longitudinal samples prior to each cycle of treatment. Patients underwent computed tomography or magnetic resonance imaging scans prior to their first treatment, and then typically at intervals of 8 weeks thereafter or when the patients were suspected with disease progression. Serum CXCL10 and SCC-Ag levels analyses were performed blinded to the radiographic changes to each patient. Ten patients who received chemoradiotherapy or chemotherapy therapy achieved partial response, as evidenced according to RECIST 1.1 measurements. Blood samples were prepared by centrifugating blood at 2053 g for 10 minutes and immediately stored at − 80 °C until analysis. Clinical pathological data including age, gender and clinical stage of healthy individuals and patients were collected. The disease was staged according to the American Joint Committee on Cancer (AJCC) TNM staging classification criteria (8th Edition). In our study, the stages of cervical cancer patients were divided into early stage including AJCC stages IA, IB, and IIA and late stage including AJCC stages IIB, III, and IV.

### Measurement of serum CXCL10 and SCC-Ag levels

The measurement of serum CXCL10 levels of all the samples was carried out by the ELISA using a commercial kit (Catalog number: DY266; R&D Systems, Minneapolis, MN, USA), following the instructions provided by the manufacturer. 96-well plates were coated overnight with 100 μl CXCL10 antibody at working concentration of 2.00 μg/ml diluted in phosphate-buffered saline (PBS). Following three washes with PBS/0.05%(w/v) Tween-20 (PBST, pH 7.4), then the plates were blocked with 300 μl blocking buffer at room temperature for 1 hour. 100 μl diluent (Blank), standard substances and serum samples (100 μl) were added and incubated for 2 hours at room temperature. 100 μl detection antibody diluted to a concentration of 20.0 ng/ml was added after washing with PBST. 100 μl streptavidin conjugated to horseradish-peroxidase secondary Ab (at1:400 dilution) was added after incubation for 2 hours at room temperature and then incubated for 20 minutes at room temperature. The plates were washed three times with PBST. Subsequently, the TMB substrate solution (Biolegend, San Diego, CA, USA) was added to each well, and the plates were incubated at room temperature. Finally, stop Solution (50 μL, Biolegend) was added to each well. The absorbance was measured using a microplate reader (Thermo Multiskan FC, VT, USA) at 450 nm with 570 nm reference. All samples were measured in duplicate.

The level of SCC-Ag in serum was measured using Abbott ARCHITECTi2000sr chemiluminescence automated immunoassay system at the department of Laboratory Medicine, Tianjin Medical University Cancer Institute and Hospital. SCC-Ag related reagents were provided by Abbott according to the manufacturer’s instructions. Based on the threshold value from the instruction of reagent company, SCC-Ag was negative when the concentration was lower than 1.5 U/ml.

### Statistical analysis

Differences of biomarker levels between groups were determined using the Mann-Whitney test. Evaluation the correlation among serum levels was done with Non-parametric Spearman’s correlation coefficients method. Receiver operating characteristic curves (ROC) were performed to assess diagnostic efficiency. The AUC (95%Cl), sensitivity and specificity were obtained from ROC analysis. The trends of the serum marker levels before and after the treatments were assessed using Wilcoxon paired two-sample test. *P* values less than 0.05 indicate statistically significant. SPSS 23.0 software (SPSS, Chicago, IL, USA) and MedCalc (version 10.4.7.0) were used for all these statistical analyses.

## Results

### Correlation between CXCL10 and clinicopathological features in CESC patients

The associations between serum CXCL10 levels and clinicopathological features of CESC patients enrolled in the present study were summarized in Table 1. Serum CXCL10 levels in patients at late tumor stage showed significant differences compared with the levels observed in patients at early tumor stage (*P* < 0.01). However, no correlation was showed between serum CXCL10 levels and age (*P* = 0.066), tumor size (*P* = 0.146), lymph node metastasis (*P* = 0.487) or distant metastasis (*P* = 0.125).

**Table 1:** Associations between serum CXCL10 levels and clinicopathological characteristics in CESC patients

Characteristics	n (%)	CXCL10 (pg/mL) Median (Interquartile range)	***P***
**Age (years)**			
≤ 51	96 (50.79)	79.18 (18.82–452.75)	0.066
> 51	93 (49.21)	88.16 (23.31–641.55)	
**Tumor size (cm)**			
≤ 4 cm	100 (52.91)	82.41 (18.82–641.55)	0.146
> 4 cm	89 (47.09)	86.67 (23.31–338.63)	
**Tumor stage**			
I-IIa	110 (58.20)	77.37 (23.31–641.55)	0.008
IIb-IV	79 (41.80)	95.82 (18.82–338.63)	
**Lymph node metastasis**			
Yes	30 (15.87)	84.10 (18.82–641.55)	0.487
No	159 (84.13)	103.09 (23.31–338.63)	
**Distant metastasis**			
Yes	9 (4.76)	83.04 (18.82–641.55)	0.125
No	180 (95.24)	145.90 (29.71–283.19)	

### Elevated serum levels of CXCL10 and SCC-Ag in CESC patients

**Fig. 1:**
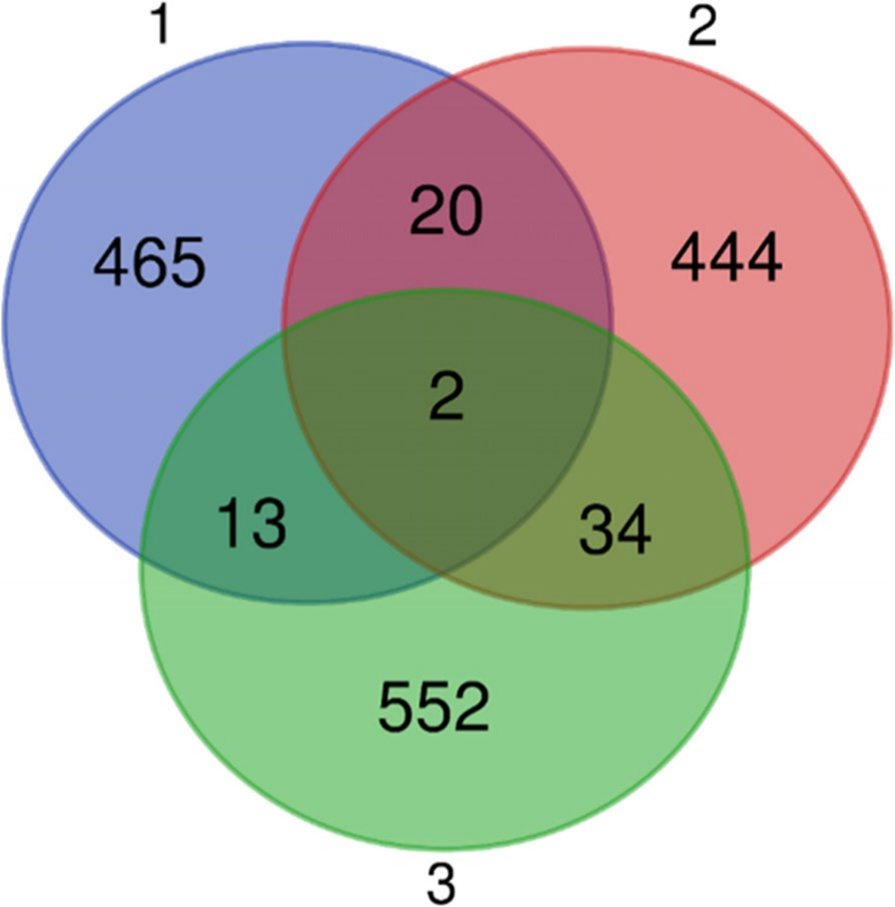
The candidate genes obtained by taking the intersection of the gene sets. (1) Top 500 upregulated expressed genes from the TCGA database. (2) Top 500 abundance genes from the TCGA database. (3) 601 secreted plasma proteins were predicted by the Human Protein Atlas database

We performed differential expression analysis between cervical cancer and non-tumor tissues. The intersection of the 500 most significantly up-regulated genes (top up-regulated genes) and 500 genes with highest TPM values (top abundance genes) in cervical cancer tissues were identified. We predicted 601 secreted plasma proteins by analysis of the Human Protein Atlas database. A total of 2 genes which encoding secreted protein were included in the intersection and selected as candidate genes (Fig. 1). Recombinant Syndecan 1 (SDC1) and CXCL10 were the two predicted genes and analysis of published works revealed that there were many reports of SDC1 in cervical cancer. Therefore, we focused on the performance of CXCL10 in this study. According to the GEPIA (http://gepia2.cancer-pku.cn/) [15], the expression of CXCL10 mRNA was significantly higher in the tissues of CESC compared with the normal tissues (Fig.S1). Analysis of published works revealed that CXCL10 involved in immune evasion which might lead to CESC tumorigenesis [14]. Therefore, we focused our attention on CXCL10 in the subsequent study.

**Fig. 2:**
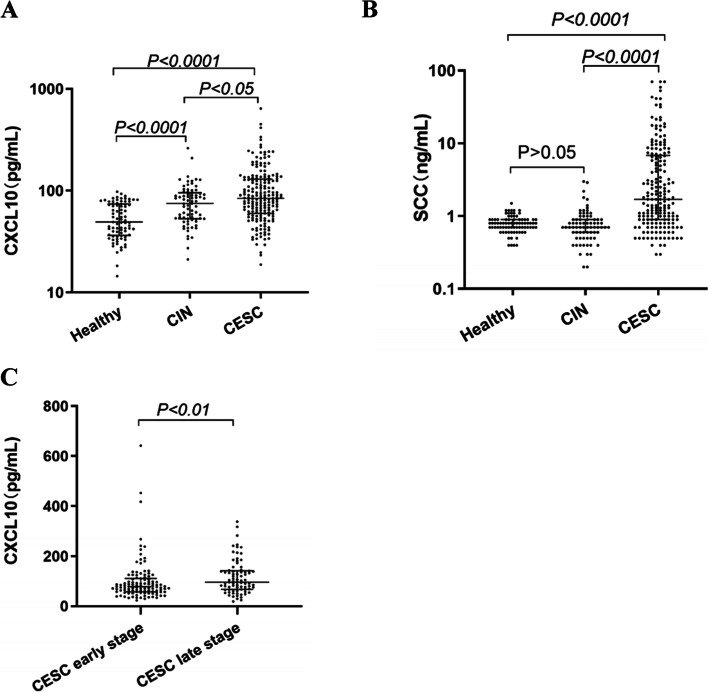
Serum levels of CXC motif chemokine 10 (CXCL10) (**A**, **C**), squamous cell carcinoma antigen (SCC-Ag) (**B**) in patients with cervical squamous cell carcinoma and control subjects. There are five groups: healthy controls (Healthy), patients with cervical intraepithelial neoplasia (CIN), patients with cervical squamous cell carcinoma (CESC), CESC early stage, and CESC late stage

Serum concentrations of CXCL10 and SCC-Ag were measured in healthy controls, patients with CIN, and patients with CESC. As shown in Fig. 2, serum levels of CXCL10 and SCC-Ag in CESC patients were significantly higher compared with those with CIN (*P* < 0.05, *P* < 0.0001) and healthy controls (all *P* < 0.0001). Serum levels of CXCL10 in CIN patients were also significantly higher than in healthy controls(*P* < 0.0001). However, there was no significant difference in SCC-Ag between the patients with CIN and the healthy controls(*P* > 0.05). Furthermore, the CESC patients were divided into two groups: CESC early stage (AJCC stages IA, IB, and IIA) and CESC late stage (AJCC stages IIB, III, and IV).

The serum levels of the two biomarkers in CESC early-stage group were also significantly elevated compared with the healthy controls (all *P* < 0.0001), however, they did not differ significantly between the patients with the CESC early stage and the patients with CIN (all *P* > 0.05). In addition, the results showed that the concentration of CXCL10 was statistically significantly higher in the late-stage group than in the early-stage group (*P <* 0.01).

### Performances of CXCL10 and SCC-Ag as individual diagnostic biomarker

**Fig. 3:**
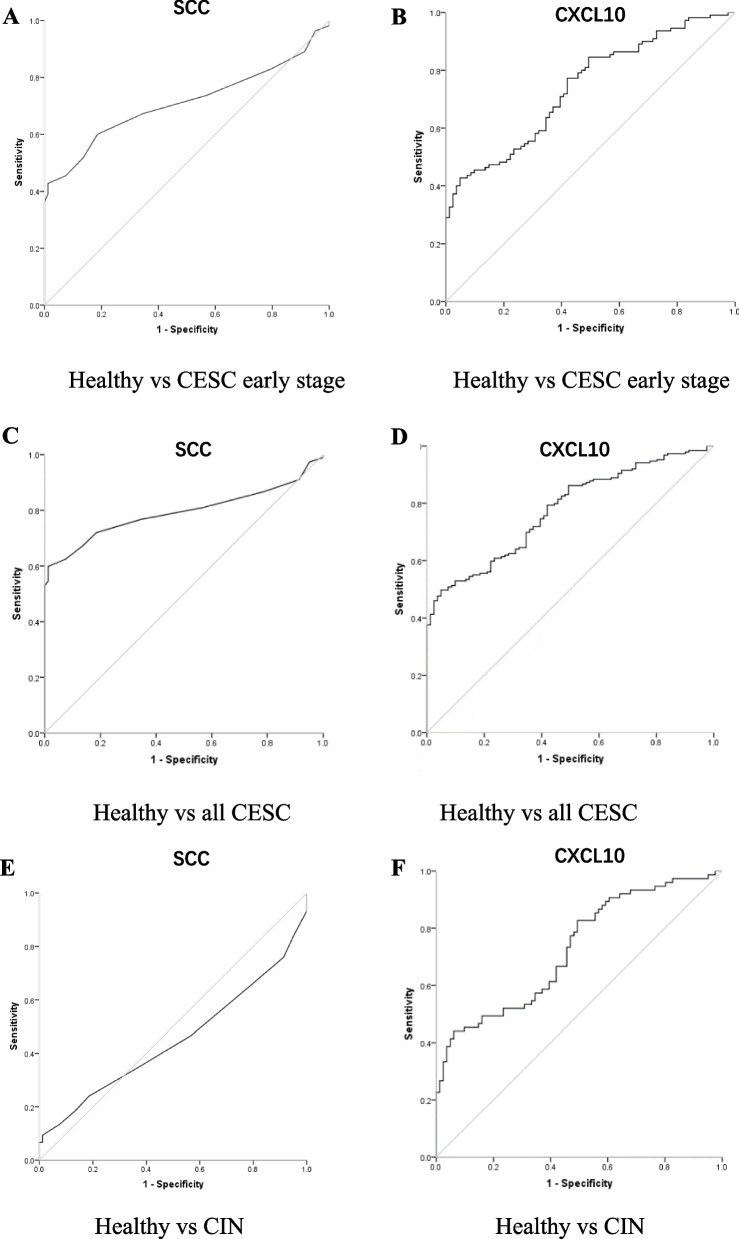
Diagnostic performances of individual CXC motif chemokine 10 (CXCL10) or squamous cell carcinoma antigen (SCC-Ag). ROC curves in distinguishing CESC early stage from Healthy controls (**A**, **B**), all CESC from healthy controls (**C**, **D**), CIN from healthy control (**E**, **F**)

**Fig. 4:**
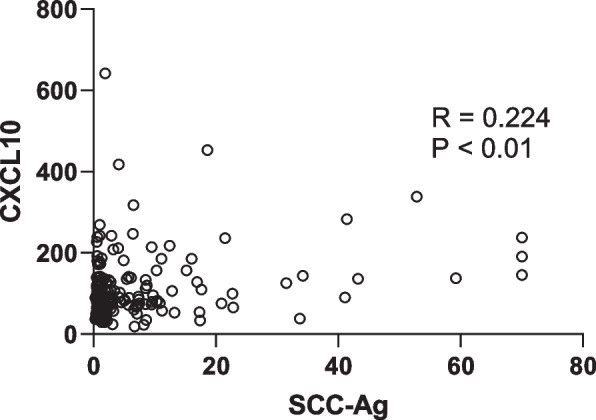
Analysis of correlation for serum CXC motif chemokine 10 (CXCL10) and squamous cell carcinoma antigen (SCC-Ag) levels in the diagnosis of cervical squamous cell carcinoma using the non-parametric Spearman’s correlation coefficients method

ROC curves were used to analyze the performances of CXCL10 and SCC-Ag as single biomarkers. As shown in the Table 2 and Fig. 3A and B, both two markers showed similar diagnostic efficacy in discriminating the early stage of CESC from healthy controls (AUCCXCL10 = 0.740, AUCSCC-Ag = 0.710, *P* > 0.05). As shown in Table 2 and Fig. 3C and D the performance of CXCL10 was comparable with SCC-Ag to discriminate CESC from healthy controls (AUCCXCL10 = 0.775, AUCSCC-Ag =0.793, *P* > 0.05). CXCL10 showed ability in distinguishing CIN from healthy control (AUCCXCL10 = 0.727) (Fig. 3F and Table 2), while SCC-Ag exhibited poor performance in distinguishing CIN from healthy control (AUCSCC-Ag =0.448) (Fig. 3E and Table 2). Non-parametric Spearman’s correlation test was used to evaluate how the two biomarkers behaved in the diagnosis of CESC. There was no correlation between serum CXCL10 and SCC-Ag (*R* = 0.224, *P* < 0 .01) (Fig. 4).

**Table 2:** Performances of biomarkers in diagnosis of cervical squamous cell carcinoma (CESC) and cervical intraepithelial neoplasia (CIN)

	AUC (95% CI)	Cutoff value	Sensitivity (%)	Sensitivity(%)	***P*** Value
**Healthy vs CESC early stage**					
CXCL10	0.740(0.671–0.809)	84.90 pg/mL	42.7	95.1	
SCC-Ag	0.710(0.637–0.784)	1.25 ng/mL	42.7	98.8	
CXCL10 + SCC-Ag	0.828 (0.771–0.884) ^*^		61.8	91.4	< 0.05
**Healthy vs all CESC**					
CXCL10	0.775 (0.719–0.830)	84.81 pg/mL	49.7	95.1	
SCC-Ag	0.793 (0.741–0.845)	1.25 ng/mL	59.8	98.8	
CXCL10 + SCC-Ag	0.877 (0.838–0.917) ^*^		74.1	92.6	< 0.05
**Healthy vs CIN**					
CXCL10	0.727 (0.648–0.805)	84.26 pg/mL	44.0	93.8	
SCC-Ag	0.448 (0.356–0.541)	1.25 ng/mL	9.3	98.8	
**Healthy vs CESC SCC-Ag-negative**					
CXCL10	0.739 (0.667–0.812)	84.90 ng/mL	42.7	95.1	

*AUC* Area under the curve, *CI* Confidence interval

^*^P < 0.05 in comparison with CXCL10 or SCC-Ag

### Combination of CXCL10 and SCC-Ag in the diagnosis of cervical cancer

**Fig. 5:**
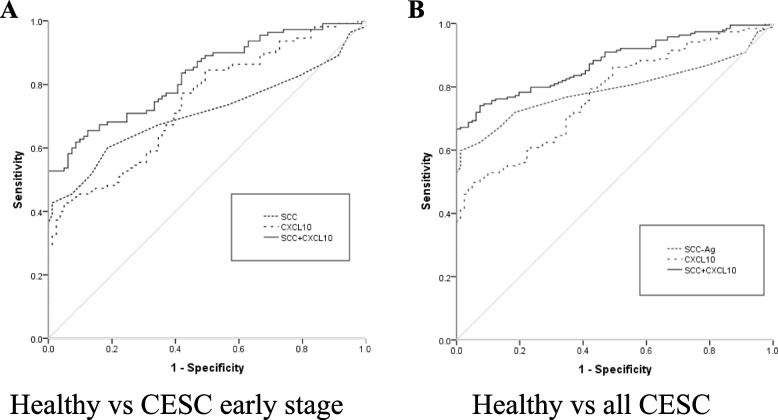
Diagnostic performances of combination of CXC motif chemokine 10 (CXCL10) and squamous cell carcinoma antigen (SCC-Ag) in the diagnosis of cervical squamous cell carcinoma (CESC), or individual CXCL10 and SCC-Ag. ROC curves in distinguishing CESC early stage from Healthy controls (**A**), all CESC from healthy controls (**B**)

To estimate the improvement in the discrimination capacity, the diagnostic efficacy of CXCL10 or SCC-Ag alone and the combination of two markers was conducted. The diagnostic efficacy of CXCL10 or SCC-Ag alone, and combined markers were compared to estimate whether the discrimination capacity of SCC-Ag could be improved by combination of the two markers. As illustrated in Fig. 5A and B and Table 2, the combined markers displayed significant improvement for AUCs than individual SCC-Ag or CXCL10 in the analysis groups (healthy vs cervical cancer early stage, healthy vs all CESC. Significant improvement for AUCs were observed to discriminate CESC early stage from healthy controls (AUCCXCL10 = 0.740, AUCSCC-Ag =0.710 vs AUCCXCL10 + SCC-Ag = 0.828, *P* < .05). The AUC was also improved 0.877(AUCCXCL10 = 0.775, AUCSCC-Ag =0.793 vs AUCCXCL10 + SCC-Ag = 0.877, *P* < .05) to distinguish healthy controls from all CESC.

### Efficacy of CXCL10 in the diagnosis of SCC-Ag-negative CESC patients

**Fig. 6:**
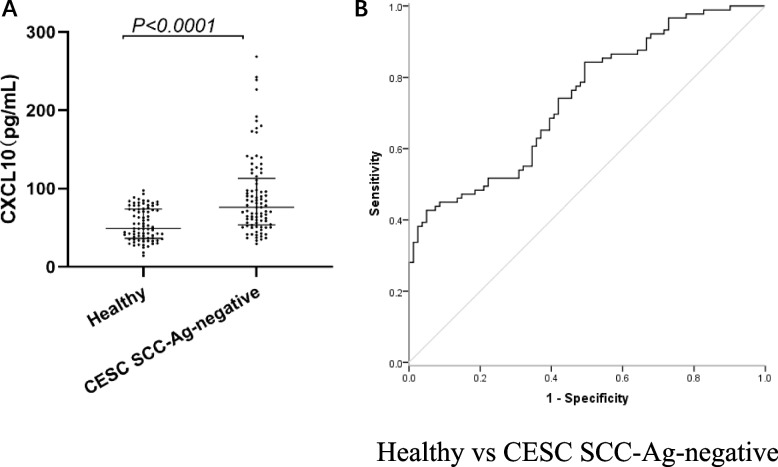
Elevated serum CXCL10 levels in patients with CESC SCC-Ag-negative. **A** Serum levels of CXC motif chemokine 10 (CXCL10) in patients with cervical squamous cell carcinoma (CESC) SCC-Ag-negative and healthy controls (Healthy). **B** Diagnostic performances of individual CXCL10 in the diagnosis of CESC SCC-Ag-negative. ROC curve in distinguishing CESC SCC-Ag-negative from healthy controls

We regarded the value of SCC-Ag lower than the threshold 1.5 ng/mL as SCC-Ag-negative. Serum levels of CXCL10 in SCC-Ag-negative CESC patients were significantly higher than in healthy controls(*P* < 0.0001) (Fig. 6A). The diagnostic ability of CXCL10 for SCC-Ag-negative CESC patients was evaluated to explore the complementary role of CXCL10 for SCC-Ag in the diagnosis of cervical cancer. CXCL10 showed ability to discriminate CESC SCC-Ag-negative from healthy controls (AUCCXCL10 = 0.739) (Fig. 6B and Table 2).

### CXCL10 and SCC-Ag dynamics of CESC patients with partial response

**Fig. 7:**
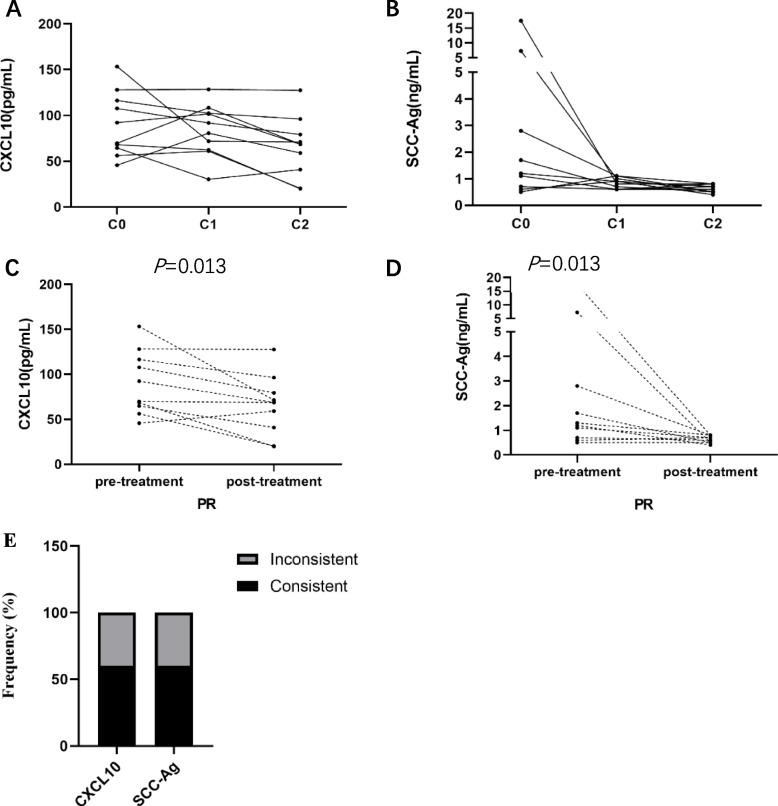
Serum CXCL10 and SCC-Ag levels of the cervical cancer patients during the treatment, who had partial response after treatment. Dynamic changes in serum CXCL10 and SCC-Ag levels (shown as concentration) (**A**, **B**). Blood samples were collected from patients prior to each time of treatment. C0 (prior to the first cycle of treatment), C1 (prior to the second cycle of treatment) and C2(prior to the third cycle of treatment). Variations in serum CXCL10 and SCC-Ag levels after two cycles of treatment (**C**, **D**). Consistent and Inconsistent frequency in changes of serum CXCL10 and SCC-Ag levels after two cycles of treatment with the partial response (**E**)

We tracked levels of CXCL10 and SCC-Ag in CESC patients during chemoradiotherapy or chemotherapy by longitudinal follow-up samples of 10 evaluable patients with partial response (PR). CXCL10 and SCC-Ag were assessed prior to each cycle of treatment and serial changes in CXCL10 and SCC-Ag after treatment were plotted (Fig. 7A, B). The cycle means the period from the initiation of one treatment course to the initiation of the next treatment course. Serum CXCL10 and SCC-Ag levels in the CESC patients significantly decreased after treatment. (*P* = 0.013, *P* = 0.013) (Fig. 7C, D). We regarded a significant relative decline (> 20%) after two cycles of treatment as consistent with the partial response to exclude the decline caused by system error of the test. The consistency was the ratio of patients whose changes of biomarker levels were consistent with the partial response. Consistent and Inconsistent frequency in serum CXCL10 levels with the partial response were the same as serum SCC-Ag levels of CESC patients who had partial response after treatment (Fig. 7E).

## Discussion

Cervical cancer is the major cause of deaths in women worldwide [1], and the survival rate is greatly reduced for patients with advanced-stage or metastasis. The diagnosis of cervical cancer is conducted by Papanicolaou test and colposcopy. Systematic screening of high-risk populations will improve poor prognosis of advanced cervical cancer. SCC-Ag is a widely used biomarker for cervical cancer with unsatisfactory diagnosis performance for detection of early-stage (AJCC stages IA, IB, and IIA) cervical cancer [16, 17].

As shown in our study, SCC-Ag showed moderate performances in some analysis groups. If the optimal cut-off was used, as many as 63(57.3%) patients of CESC early stage and 76(40.2%) patients of CESC would be falsely considered as healthy. Additionally, there was no difference of SCC-Ag between healthy control and CIN patients. Therefore, it is necessary to find a new effective biomarker for CESC or to combine with SCC-Ag to improve the diagnostic performance for CESC. In the present study, we found that CXCL10 could play complementary role for SCC-Ag as a potential biomarker in prediction of early CESC.

CXCL10 is a CXC chemokine family protein whose widely known function is participating leukocyte trafficking. Moreover, it also plays important roles in numerous cancers. Studies have shown that CXCL10 has dual effects on tumor progression. It has been reported CXCL10 exert anti-malignancy function by inhibiting angiogenesis and influencing tumor microenvironment [18, 19]. However, emerging reports focused on tumor-promoting ability of CXCL10 by increasing cell proliferation and metastasis [20, 21]. CXCL10 mRNA is highly expressed in various tissues including CESC [22]. However, there was no study have accessed the clinical value of serum CXCL10 in patients with CESC. In this study, we focused on diagnostic value of CXCL10 as a serum biomarker for CESC. Our results showed that serum CXCL10 levels in patients with CESC were significantly upregulated compared with healthy controls, which was consistent with the results from TCGA database that levels of CXCL10 mRNA increased in CESC tissues compared with normal tissues. It has been reported that CXCL10 mRNA was greatly upregulated both in cervical tissues of HPV infected patients with CIN or CESC. Moreover, it has been also demonstrated that CXCL10 and its receptor CXCR3 were expressed by cervical cancer cells [23]. The clinically used cutoff for SCC-Ag is 1.5 ng/ml according to the instruction from the manufacture. The optimal threshold in this study was less than 1.5 ng/ml, which may be due to the different population included in different studies. Elevations are also observed in SCC malignancies, such as the head and neck, esophagus, skin, lung. There was no other study of CXCL10 levels in cervical cancer patients. The ranges of the circulating CXCL10 detected in other study on breast cancer were different from the values in our study. This can be explained by different reagents, expression of CXCL10 in different cancers and the exclusion criteria of the healthy controls in different studies.

These results indicated that CXCL10 was a potential marker of CESC. As an individual biomarker, CXCL10 showed moderate diagnostic performance which was comparable with SCC-Ag, as indicated by the AUC. CXCL10 would not be considered to replace SCC-Ag for diagnose CESC. We investigated the supplemental performance of CXCL10 for SCC-Ag in the early diagnosis of CESC in our study. Obvious improvements were observed in the diagnostic abilities of SCC-Ag for CESC combining with CXCL10, as well as in the diagnosis of CESC early stage, which may be explained by the results that there was no correlation between serum CXCL10 and SCC-Ag. Furthermore, in our study, the serum levels of CXCL10 retained moderate diagnosis capabilities for patients with SCC-Ag-negative CESC. All these results indicated that CXCL10 might provide an essential complement for SCC-Ag. All in all, these data illustrated that the combination of CXCL10 and SCC-Ag was an effective diagnosis biomarker for CESC.

CIN may develop slowly to invasive cervical cancer, so that it is important to detect disease and intervene the progression [24]. As shown in our study, although SCC-Ag had the diagnostic abilities in some analysis groups, it showed no ability in distinguishing CESC SCC-Ag-negative from healthy controls or CIN from healthy controls. However, serum levels of CXCL10 in CIN patients were substantially higher than those in healthy controls, and serum levels of CXCL10 in CESC patients were significantly elevated compared to those in CIN patients. The mechanism for the results has not been completely clear, however it might be explained by the previous report that CXCL10 increased significantly in cervical tissues of HPV infected patients with CIN which might progress into invasive carcinoma and CXCL10 participated in the progression of carcinogenesis [23]. In this study, serum CXCL10 levels after treatment decreased significantly, and serum CXCL10 concentrations at baseline were higher in more advanced tumor stages. One possible explanation for these results might be that the cancer tissues were the major source of serum CXCL10 in patients with CESC. CXCL10 showed moderate diagnostic performance as individual serum CESC marker in distinguishing both CESC patients from control subjects and early-stage CESC patients from control subjects. The results that serum CXCL10 concentrations of the patients with advanced CESC were higher than those with early stages of CESC suggested CXCL10 took apart in the process of CESC progression because tumors with late stages were usually cancers with metastases. Our study concluded serum SCC-Ag and CXCL10 were independent from each other which suggested they might play different roles in CESC progression.

Previous studies have shown that CXCL10 is a potential tumor biomarker of malignant diseases which not only has diagnostic value but also has prognostic value for tumors, such as hepatocellular carcinoma, colorectal cancer, and breast cancer [13, 20, 25]. And no previous studies were related to whether high levels of serum CXCL10 were associated with poor prognosis of patients with CESC. In this study, we mainly focused on discovering and validating biomarker for cervical cancer detection, not refer to prognosis. The result of our study that serum CXCL10 concentrations of patients with late-stage (AJCC stages IIB, III, and IV) CESC were higher than those with early-stage CESC suggested CXCL10 might take part in the progression of CESC and be a prognostic marker of the CESC patients. We need to conduct longitudinal follow-up study of CESC patients to explore the prognostic role of serum CXCL10 levels. There were few previous studies have mentioned the performance of CXCL10 in assessing curative effect. In this study, we tracked the dynamics of serum CXCL10 levels in 10 CESC patients receiving treatment of chemoradiotherapy or chemotherapy. We found that the serum CXCL10 levels in CESC patients with PR response after treatment were significantly different from levels prior to treatment. It is further validation of that CXCL10 is a diagnostic biomarker of cervical cancer. Previous studies reported SCC-Ag was valuable in monitoring of curative efficacy of CESC patients [26, 27]. Moreover, the consistence of changes in CXCL10 levels with PR response was the same as in SCC-Ag levels after treatment, which suggested that CXCL10 might reflect the curative efficacy of CESC patients comparably with SCC-Ag. The samples for accessing treatment response in this study were collected from the patients underwent chemoradiotherapy or chemotherapy. It is necessary to enlarge the sample size and analyze the changes of serum CXCL10 levels before and after surgery to further explore the clinical value of CXCL10 in the curative efficacy of cervical cancer.

In conclusion, our study firstly fully illustrated the potential value of serum CXCL10 as a diagnostic biomarker for CESC. The performance of combination of CXCL10 and SCC-Ag was fully investigated in this study. Serum CXCL10 levels may be a novel and useful predictor for CESC as well as it can improve the diagnostic efficiency of SCC-Ag in prediction of cervical cancer. CXCL10 was still valuable in the diagnosis of SCC-Ag negative CESC. The combination of CXCL10 and SCC-Ag showed significantly improved performance compared with SCC-Ag alone. In conclusion, this study indicated that CXCL10 was a potential serum biomarker as a supplement to SCC-Ag in diagnosing cervical cancer.

## Supplementary Information

**Additional file 1.**

## Data Availability

All data supporting the conclusions of this article are included in the article.

## Abbreviations

CXCL10: CXC motif chemokine 10
SCC-Ag: Squamous cell carcinoma antigen
CESC: Cervical squamous cell carcinoma
CIN: Cervical intraepithelial neoplasia
AJCC: American Joint Committee on Cancer
PBS: Phosphate-buffered saline
PBST: Phosphate-buffered saline/0.05%(w/v) Tween-20
GEPIA: Gene Expression Profiling Interaction Analysis
TCGA: The Cancer Genome Atlas
ROC: Receiver operating characteristic curve
ELISA: Enzyme-linked immunosorbent assay
AUC: Area under the curve
