# Supplementary figures and images for: CXCL10 serves as a potential serum biomarker complementing SCC-Ag for diagnosing cervical squamous cell carcinoma
Source: BMC Cancer. 2022 Oct 8;22:1052. doi: 10.1186/s12885-022-10142-0 (PMC9547472; doi:10.1186/s12885-022-10142-0)

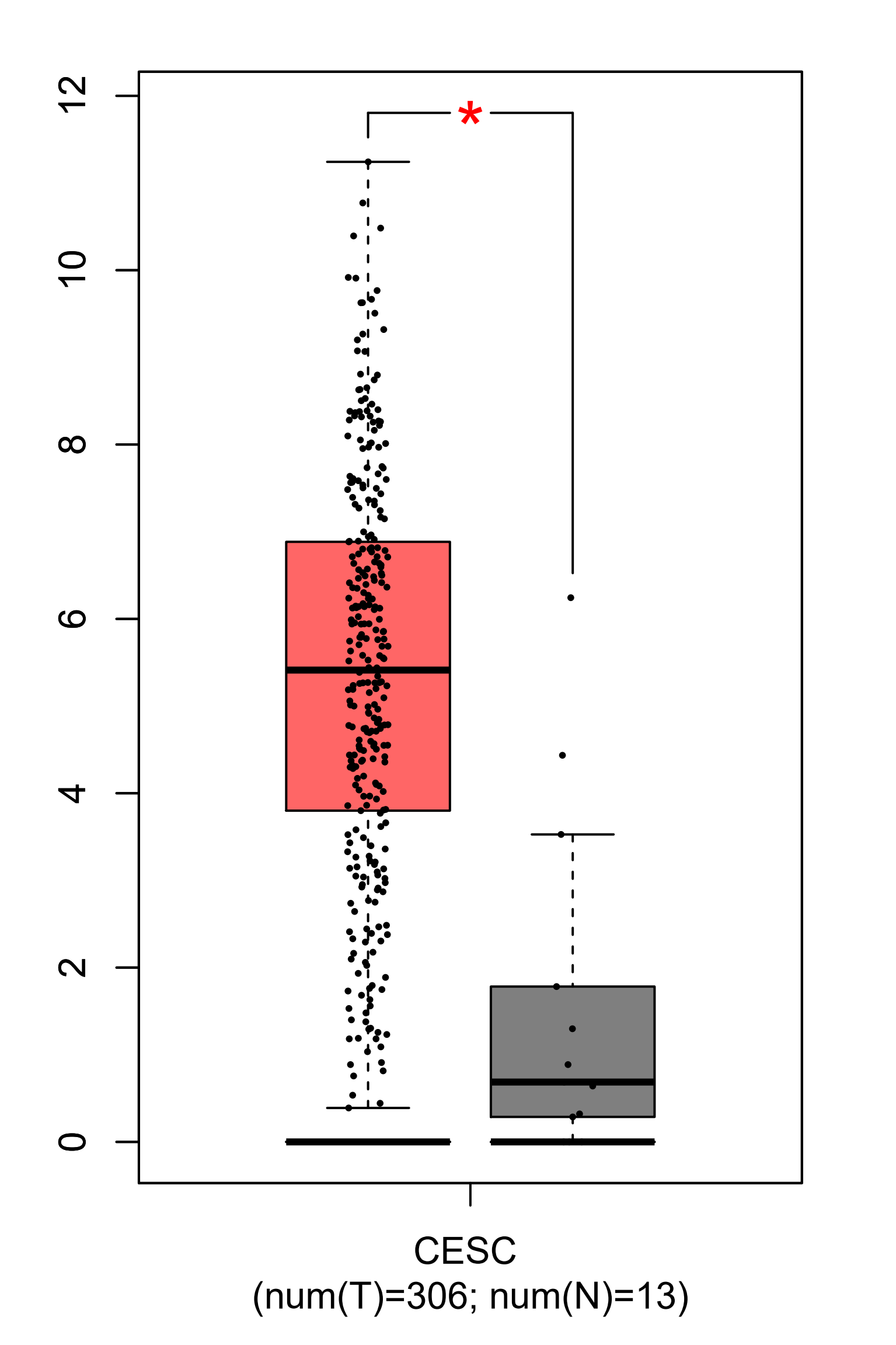


Fig. S1 The mRNA expression of CXCL10 in cervical cancer and normal tissues from GEPIA.

Supplement: Supplementary file 1 — Additional file 1. [file 12885_2022_10142_MOESM1_ESM.docx]
